# Investigation of the anti-cancer effect of quercetin on HepG2 cells in vivo

**DOI:** 10.1371/journal.pone.0172838

**Published:** 2017-03-06

**Authors:** Jin Zhou, Li Fang, Jiaxu Liao, Lin Li, Wenxiu Yao, Zhujuan Xiong, Xiang Zhou

**Affiliations:** 1 Department of Chemotherapy, Sichuan Cancer Center, School of Medicine, University of Electronic Science and Technology of China, Chengdu, Sichuan, P.R. China; 2 Department of Gastroenterology, the First Affiliated Hospital, Chengdu Medical College, Chengdu, Sichuan, P.R. China; 3 Department of Radiology, the Sixth Hospital, Chengdu, Sichuan, P.R. China; 4 Department of Nuclear Medicine, the Second Hospital, Chengdu, Sichuan, P.R. China; Istituto Superiore di Sanità, ITALY

## Abstract

Quercetin, a natural polyphenolic flavonoid compound, can inhibit the growth of several malignant cancers. However, the mechanism still remains unclear. Our previous findings have suggested that quercetin can significantly inhibit HepG2 cell proliferation and induce cell apoptosis *in vitro*. It can also affect cell cycle distribution and significantly decrease cyclin D1 expression. In this study, we investigated the anti-cancer effect of quercetin on HepG2 tumor-bearing nude mice and its effect on cyclin D1 expression in the tumor tissue. First, the nude murine tumor model was established by subcutaneous inoculation of HepG2 cells, then quercetin was administered intraperitoneally, and the mice injected with saline solution were used as controls. The daily behavior of the tumor-bearing mice was observed and differences in tumor growth and survival rate were monitored. The expression of cyclin D1 in isolated tumor sections was evaluated by immunohistochemistry. We found that HepG2 tumor became palpable in the mice one-week post-inoculation. Tumors in the control group grew rapidly and the daily behavior of the mice changed significantly, including listlessness, poor feeding and ataxia. The mice in quercetin-treated group showed delayed tumor growth, no significant changes in daily behavior, and the survival rate was significantly improved. Finally, we observed increased tumor necrosis and a lighter cyclin D1 staining with reduced staining areas. Our findings thus suggest that quercetin can significantly inhibit HepG2 cell proliferation, and this effect may be achieved through the regulation of cyclin D1 expression.

## Introduction

Hepatic cancer is one of the most common malignant cancers in China and it seriously threatens people’s health. The onset of hepatic cancer is usually occult and difficult to be diagnosed at the early stage, but it grows rapidly and shows the high rate of metastasis. As a result, most patients are diagnosed with hepatic cancer at the advanced stages. Currently, the most common treatments are surgery, stereotactic radiation, chemotherapy and interventional embolization, as well as the recently developed targeted cancer therapy. However, in most cases, therapeutic outcomes are not ideal [1]. Hepatic cancer is a polygenic disease with complex mechanism and signaling pathways, for which it is difficult to achieve a successful therapeutic effect by single treatment modality. Therefore, the use of targeted agents to regulate multiple signaling pathways has emerged as a novel paradigm for hepatic cancer treatment.

Quercetin, a natural flavonoid found in plants, has been extensively investigated for its biological activities. Quercetin shows significant inhibition effects on malignant cells growth in leukemia, breast, hepatic, ovarian, colorectal, gastric, and endometrial cancers [2–4]. Several studies have shown that quercetin controls cancer cell growth through the regulation of specific signaling pathways, such as decreasing oncogene expression, inducing malignant cells apoptosis and inhibiting angiogenesis, etc. [5,6]. Our previous studies showed that quercetin can significantly inhibit the proliferation of HepG2 cells and induce apoptosis, possibly through the participation of cyclin D1 regulation [7]. However, these results were obtained by using an *in vitro* system. To further investigate the anti-hepatic cancer effect of quercetin *in vivo*, we established a subcutaneous HepG2 tumor-bearing nude murine model and assessed the effect and mechanism of quercetin on tumor growth.

## Materials and methods

### Materials

Quercetin (PH1488) was purchased from SIGMA (St. Louis, MO, USA). It was first dissolved in DMSO and then diluted in saline (final DMSO content <0.1%) before administration. HepG2 cell line was purchased from ATCC (Manassas, VA, USA). Balb/c/nu mice were purchased from Charles River Laboratories (Beijing, China). Cyclin D1 antibody was purchased from Santa Cruz Biotechnology (Santa Cruz, CA, USA). Fetal bovine serum, Dulbecco's Modified Eagle's medium (DMEM) and trypsin were from Yubo Biotech Ltd (Shanghai, China). Animal care followed the guidelines of the Sichuan Animal Care Committee and all study protocols were approved by the Sichuan Cancer Hospital Ethics Committee.

### Cell culture and tumor inoculation

HepG2 cells were cultured in 5% CO_2_ at 37°C in DMEM after resuscitation. Then we passaged cells continuously. Right before inoculation, HepG2 cells in the logarithmic growth phase were harvested and re-suspended at a final concentration of 6×10^7^/mL? BALB/c/nu female mice, 6–8 weeks of age (body weight 17±2 g) were randomly caged. 75% ethanol was used for the disinfection of the skin at the right lower flank of the mouse before the inoculation and then 6×10^6^ cells in 100 μL were injected subcutaneously in each mouse. The mice were randomly grouped as the treatment group and the control group (25 in each group) when tumor volume reached 200 mm^3^ (n = 10 for tumor growth and survival study and n = 15 for tumor histological examination) and the mice with no tumor growth were removed from study.

### Drug administration and *in vivo* tumor volume monitoring

Diluted quercetin in DMSO solutions were intraperitoneally administrated at 10 mg/kg for the seven consecutive days [8]. Saline with the same volume was injected into the mice of the control group. Tumor volume was measured every three days using a digital caliper and calculated using the following formula:

$$\mathrm{Tumor}\;\mathrm{volume}\;\left(mm^{3}\right)\;V=0.5\times a\times b^{2}$$

Where a and b represent the maximum width and length of the tumor respectively [9].Tumor growth curve was drawn and the mouse survival rate was recorded. Five mice were sacrificed by cervical dislocation on day 7, 14 and 21. Tumors were excised using ophthalmic forceps and scissors. The tumor tissue sections less than 5 mm in thickness were fixed in pre-labeled containers with 4% paraformaldehyde for at least 24 hrs(hours?) for immunohistochemistry studies.

### Histological examination and immunohistochemistry staining

The tissue paraffin sections (4 μm in thickness) were stained with hematoxylin and eosin (H&E) and immune-stained for cyclin D1 expression. Cyclin D1 antibody was diluted 1:30 and incubated with the tissue section at 4°C overnight. The sections were then incubated with a secondary antibody, followed by DAB staining, hematoxylin staining, dehydration and mounting. The immune-stained sections were then analyzed onto a XY microscope by using the DM2500 image analyzing software.

The immunohistochemistry results were assessed using a two-parameter semi-quantitative method. Five continuous microscopic field areas were scanned from each section. The positive rate of stained cells and the staining intensity were examined and scored under high magnification. The percentage of the positively stained HepG2 cells within each microscopic field was scored on a 1–4 scale (1 = 0–25% positive cells, 2 = 6–50% positive cells, 3 = 51–75% positive cells, and 4 = 76–100% positive cells). The stain intensity was scored on a 0–3 scale (0 = no color, 1 = light yellow, 2 = yellow-brown, and 3 = dark brown). The scores of the positive rate of stained cells and the stain intensity were added to obtain a composite score. All slides were blinded and the assessment was performed by the same pathology doctor.

### Statistical analysis

Statistical analysis was performed using IBM SPSS version 19.0 software. Differences among groups were analyzed using single factor variance analysis; comparison was assessed by unpaired t test. Mouse survival data was calculated using Kaplan-Meier method and the statistical analysis of the survival rate was performed using Log-rank test. Only results with p<0.05 were considered statistically significant.

## Results

### Behavior studies of the HepG2 tumor-bearing BALB/c/nu mice

The HepG2 tumor cells were inoculated as described in the method section. A total of 60 mice were subcutaneously inoculated. Then, 7 mice did not form subcutaneous tumor. Besides, other 3’s subcutaneous tumors were too small to reach the standard. So a total of 10 mice were removed. The remaining 50 were vaccinated according to the experimental design. The tumors became palpable one-week after cells inoculation. The mice in the control group showed rapid tumor growth and significant changes in their daily behaviors, presenting listlessness, poor eating and ataxia. Mania and hyperactivity were observed in some cases. Compared to the control group, the mice treated with quercetin exhibited slow tumor growth rates and no significant behavioral changes.

### Comparison of the tumor growth rates and survival curve of the HepG2 tumor-bearing BALB/c/nu mice

**Fig 1:**
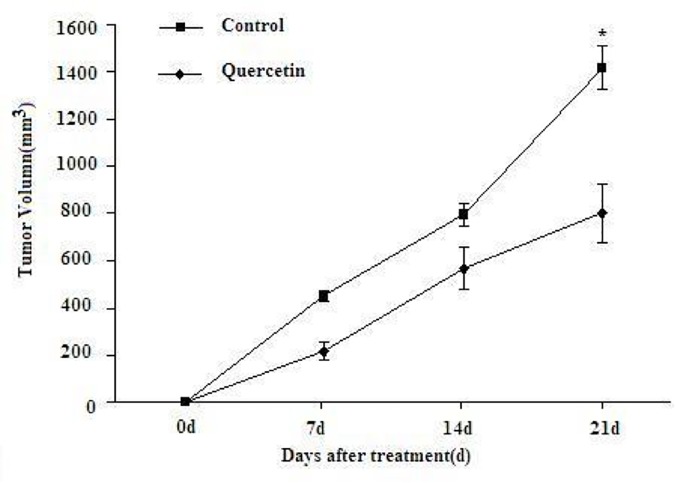
Tumor growth curves of the subcutaneous HepG2 tumor-bearing mice. Compared to the control group, the mice in the quercetin-treated group showed delayed tumor growth and a smaller tumor volume (p < 0.05).

**Fig 2:**
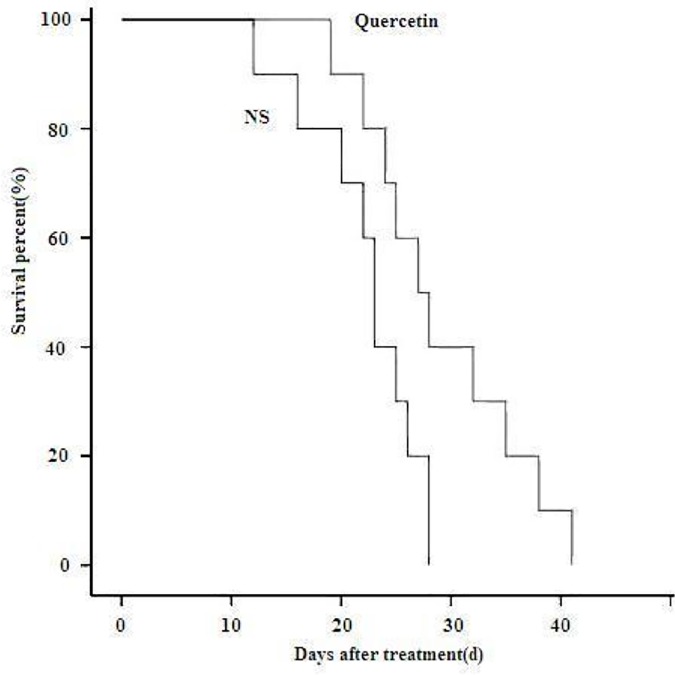
Survival curves of the subcutaneous HepG2 tumor-bearing mice. Compared to the control group, mice in the quercetin-treated group showed prolonged survival rate.

The mice in the control group showed a more rapid tumor growth rate compared to the mice in the quercetin-treated group, in which process the tumor growth was significantly inhibited and relatively smaller tumor volumes were observed. The tumor growth curves corresponding to the different treatments are reported in Fig 1, and the difference between the two groups was statistically significant. In the animal survival study, the mice in the control group started to die on day 12, whereas quercetin-treated mice on day 20. All mice from the control group were dead on day 28 while mice in the treated group survived till day 41. The survival curves are shown in Fig 2.

### Tumor histological examination

**Fig 3:**
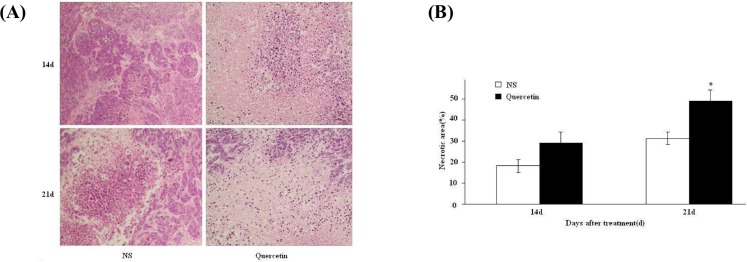
Hematoxylin and eosin (H&E) staining for the histological examination of the inoculated subcutaneous tumor. A, H&E histological images of the tumor tissues on day 14 and 21 post-treatment (200X magnification); B, Comparison of tumor necrotic areas between the quercetin-treated and the control groups (*p < 0.05).

In both control and treated groups, tumors were excised and inspected by the histological staining with hematoxylin and eosin. In the control group, the tumor cells divided actively with the visible necrotic area. In the quercetin-treated group, he cell mitosis was reduced with the increased and connected tumor necrotic areas. The difference in the proportion of the necrotic areas between the two groups was statistically significant (Fig 3A and 3B).

### Immunohistochemistry evaluation of cyclin D1 expression

**Fig 4:**
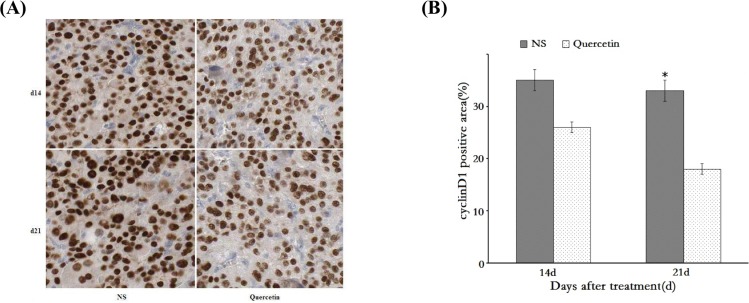
Immunohistochemistry analysis of cyclin D1 expression in subcutaneous tumor tissues. A: Immunohistochemistry images of cyclin D1 expression in subcutaneous tumor tissues; B: Comparison of the cyclin D1 positive areas between the quercetin-treated and control groups (*p < 0.05).

In our previous *in vitro* experiments, quercetin showed a significant effect on inhibiting the expression of the cell cycle regulation gene cyclin D1. In the present *in vivo* study, we observed that quercetin showed a significant negative regulatory effect on the growth of the inoculated HepG2 tumors. We thus conducted the immunohistochemistry analysis to compare the differences in cyclin D1 expressions before and after quercetin treatment. As shown in Fig 4A, the nucleus of tumor cells was positively stained. Compared to the control group, cyclin D1 staining in the tumor cells of quercetin-treated group exhibited a statistically significant decrease in intensity and the reduction on the percentage of positively stained cells (Fig 4B). The comparison of the immunohistochemistry scoring of the two groups is summarized in Table 1.

**Table 1:** Comparison of the immunohistochemistry scoring of cyclin D1 expression in HepG2 tumor tissues between the quercetin-treated and control groups (control vs quercetin, *p< 0.05).

	0	7d	14d	21d
control	6.27±0.26	6.09±0.34	6.17±0.39*	5.91±0.22*
quercetin	6.19±0.31	5.47±0.21	4.59±0.17*	3.82±0.16*

## Discussion

Malignant tumors represent one of the major threatens people's health. In China, hepatic cancer is the malignant tumor with the highest incidence, sustained also by the high incidence of hepatitis. Hepatic cancer is usually occult in onset and it is difficult to be diagnosed at the early stage but it grows fast with a high metastasis rate. For the majority of the patients, the diagnosis are often made when the best opportunities for treatment are missed, thus evolving into undesirable outcomes. Hepatocellular carcinoma accounts for 90% of liver cancers. Currently, the most commonly used clinical treatments are surgery, stereotactic radiation, chemotherapy and interventional embolization, as well as the recently developed targeted cancer therapy. However, in the most cases, therapeutic outcomes are not successful. The clinical benefits from the new treatment approaches are far less than the problems so far [1]. Cancer is a polygenic disease involving complex mechanisms and signaling pathways, and therefore it is difficult to achieve a successful therapeutic effect by one single treatment modality. In addition, drug resistance and the re-activation of alternative signaling pathways are the main reasons for cancer relapse or metastasis. As a result, the current anti-cancer drug development and research are focused on the extended pharmacological effects and low toxicity therapeutic agents.

Flavonoids are a class of naturally occurring compounds widely distributed as polyphenol pigments in fruits, flowers, vegetables, and cereals. Flavonoids are also known as bioflavonoids. Along with their derivatives, they show a variety of biological activities, including oxygen free radical removal, anti-inflammatory, anti-oxidation, anti-tumor, anti-viral, blood lipid regulation and control of blood glucose [10–12]. According to the structural differences, the oxidation degree of the central three-carbon chain and the difference in the B ring coupling position, flavonoids compounds can be divided into flavonols, isoflavones, flavonoids and other 14 categories [13]. Quercetin, which belongs to the flavonol category, is a typical representative of the flavonoids. In addition to general pharmacological activities, quercetin exhibits important negative regulatory effects on the proliferation of various cancer cells. In many *in vitro* and *in vivo* studies reported in recently years, quercetin can significantly inhibit the growth and proliferation of several types of cancer, including breast, liver, nasopharyngeal, colorectal, gastric, endometrial cancers, and leukemia. Quercetin exerts its biological functions with the low systemic toxicity, thus attracting attentions from researchers [2–4].

Quercetin has a wide range of activities involving several molecular pathways and mechanisms. It has been reported that quercetin can inhibit the expression and activity of androgen receptor (AR) through Sp1-mediated blockage of c-Jun N-terminal kinase (JNK) signaling pathway, therefore reducing the invasiveness of prostate cancer cells [14]. Previous studies demonstrated that quercetin is able to down-regulate cell cycle genes and up-regulate anti-cancer genes as p27 *via* Wnt signaling pathway, and inhibit tumor growth [15,16]. Quercetin can also inhibit tumor angiogenesis. Some studies suggested a reduced neo-vessel density and lower VEGF expression in tumor-bearing mice treated with quercetin [17].

Drug resistance tends to significantly affect the cancer chemotherapeutic outcomes and it is mostly associated with the expression of P-glycoprotein (P-gp), which is responsible for the phenomenon of multidrug resistance. In previously reported *in vitro* studies, quercetin down-regulated the expression of P-gp, therefore increasing the sensitivity of gastric cancer cells to anti-cancer agents [18]. Shen *et al*. [19] reported that for the drug resistant cells induced by thermal therapy, if quercetin was given in advance, it could significantly enhance the reverse activities, thus increasing the sensitivity of the tumor cells.

In our previous study, we evaluated the effect of quercetin on HepG2 cells. After labeling of leucine with stable isotope ^2^H in the HepG2 cells, the cells were treated with quercetin and assessed by mass spectrometry (MS). The results showed that quercetin could affect the expression of various functional proteins involved in specific signaling pathways. We validated MS results by Western blot analysis, by which we identified the cell adhesion -associated protein IQGAP1 as an important key factor, and speculated that this might be one of the quercetin’s anti- HepG2 cell migration mechanisms [20]. In addition, the low expression of cyclin D1 was observed. Later, we conducted *in vitro* experiments and observed changes in HepG2 cell cycle after the administration of quercetin [7]. Therefore, the focus of this study was the *in vivo* anti-cancer effects of quercetin on HepG2 tumor bearing animal model.

Cyclin D1 is a subtype of cell cycle protein cyclin D. Compared to the other two subtypes D2 and D3, the study of cyclin D1 is relatively adequate. Cyclin D1 contains 295 amino acids, and the sequence comprising amino acids 56–114 is the main structure. Cyclin D1 shows short plasma half-life, which is less than 25 min. The expression of cyclin D1 is cell cycle dependent and reaches the maximum in mid of G1 phase. The main function of cyclin D1 is to maintain cell cycle and to promote cell proliferation. Cyclin D1 can bind with cyclin-dependent kinase (CDK4), phosphorylate the inhibiting protein retinoblastoma (Rb) in G1 period, which leads to the disassociation of Rb from E2F transcription factor, trigger mRNA transcription and promote G1/S transition [21]. As a result, the abnormal expression of cyclin D1 can significantly alter the cell cycle and induce disorder in cell proliferation. Cyclin D1 can also promote gene transcription through histone deacetylase P/CAF and transcription factor TFII-D [22]. Cyclin D1 is considered as a proto-oncogene due to its promotion effect on cell proliferation. Its over-expression may lead to rapid cell growth and malignancy. So far, several studies have demonstrated that high cyclin D1 expression was observed in cancers including breast, lung, prostate, lymph node and colorectal cancers [23–25].

The overexpression of cyclin D1 is associated with the therapeutic outcomes. A large? (large scape of) study shows that in ER-positive breast cancer, the overexpression of cyclin D1 was significantly associated with fast metastasis and shortened lifespan [26]. Cyclin D1 overexpression may also lead to the resistance of various therapeutic agents such as cytotoxic drugs, estrogen antagonists, tyrosine kinase inhibitor Gefitinib, as well as BRAF and MEK signaling pathway inhibitors [27]. In addition, cyclin D1 is also involved in the production of acquired radiation antibodies [28]. It has been suggested that quercetin can regulate the expression of P-gp, and therefore, reverse multi-drug resistance. Combining the effect of cyclin D1 in drug resistance, the interaction of the two signaling pathways should be investigated in further research.

Our *in vitro* studies showed that quercetin could significantly inhibit proliferation, induce apoptosis and alter cell cycle in HepG2 cells. It also reduced the gene expression of cyclin D1 in HepG2 cells. In the present *in vivo* study based on the use of a nude mouse model, we showed that in mice treated with quercetin the tumor growth was significantly delayed and animal survival was prolonged in comparison with the control group. This study was a subcutaneous tumor model. However, even though subcutaneous implants are growing rapidly, this treatment does not only have a great impact on the life habits and daily activities of mice, but also have the potential effects on the changes of the internal organs and the body environment. After 12 days of inoculation, the mice in the control group began to die. In the treatment group, quercetin delayed the development of tumor. At the beginning of the twentieth day, the death of the mice in quercetin group was observed. Tumor histological examination and immunohistochemistry showed an increase in tumor necrosis and reduction in intensity and percentage of the positive areas of cyclin D1 staining. Combining these results and those obtained with the *in vitro* analysis, we can conclude that quercetin could significantly inhibit the growth and proliferation of HepG2 cells by regulating the gene expression of cyclin D1.

## Supporting information

S1 Fig Animal model. (JPG)

S1 Table Immunohistochemical analysis. (XLSX)

S2 Table Survival curves. (XLS)

S3 Table Tumor volume curves. (XLS)
