# Supplementary figures and images for: Investigation of the anti-cancer effect of quercetin on HepG2 cells in vivo
Source: PLoS One. 2017 Mar 6;12(3):e0172838. doi: 10.1371/journal.pone.0172838 (PMC5338765; doi:10.1371/journal.pone.0172838)

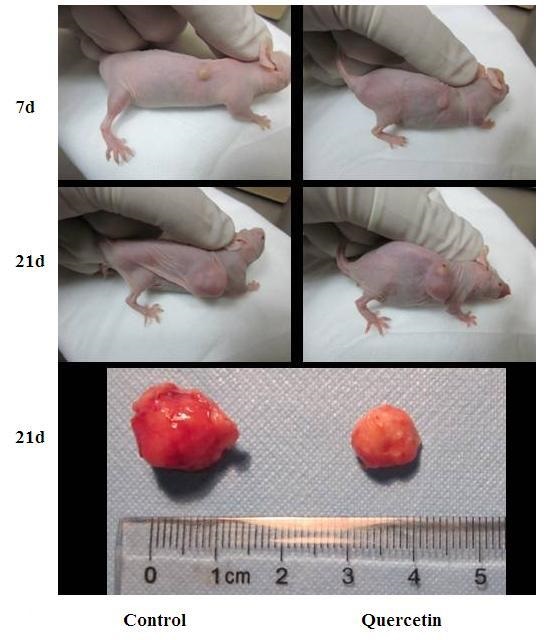

Supplement: S1 Fig — (JPG) [file pone.0172838.s001.jpg]
